# Impact of Dexmedetomidine-Based Opioid-Sparing Anesthesia on Opioid Use After Minimally Invasive Repair of Pectus Excavatum: A Prospective Randomized Controlled Trial

**DOI:** 10.3390/jcm13237264

**Published:** 2024-11-29

**Authors:** Minju Kim, Jaewon Huh, Hoon Choi, Wonjung Hwang

**Affiliations:** Department of Anesthesiology and Pain Medicine, Seoul St. Mary’s Hospital, College of Medicine, The Catholic University of Korea, Seoul 06591, Republic of Korea; jkkpsk@gmail.com (M.K.); ether@catholic.ac.kr (J.H.); hoonie83@catholic.ac.kr (H.C.)

**Keywords:** dexmedetomidine, opioid, opioid-free anesthesia, opioid-sparing anesthesia, pectus excavatum

## Abstract

**Background:** Opioid-sparing anesthesia (OSA) using dexmedetomidine has gained attention as an alternative to opioid-based anesthesia (OBA) due to its potential to reduce opioid consumption and the associated side effects. This study aimed to investigate the effect of dexmedetomidine-based OSA on postoperative pain intensity, opioid consumption, and recovery outcomes in patients undergoing a minimally invasive repair of pectus excavatum. **Methods:** Eighty-four patients undergoing a minimally invasive repair of pectus excavatum were randomized to either the OSA group, receiving dexmedetomidine, or the OBA group, receiving remifentanil. The primary outcome was the total amount of analgesics administered within 24 h postoperatively. The secondary outcomes included pain intensity and analgesic consumption over 48 h, recovery outcomes, intraoperative hemodynamics, and opioid-related complications. **Results:** The OFA group reported a significantly reduced total morphine-equivalent dose within 24 h (55.4 ± 31.1 mg vs. 80.2 ± 26.7 mg, *p* < 0.001) and lower VAS scores at 24 h (3.9 ± 1.5 vs. 5.4 ± 2.1, *p* < 0.001). Pain intensity was lower, and analgesic consumption was reduced in the OSA group 1–6, 6–24, and 24–48 h after surgery. Recovery times and intraoperative hemodynamics were comparable between the groups, with no significant differences in opioid-related complications. **Conclusions:** Dexmedetomidine-based OSA effectively reduces postoperative pain and opioid use without compromising recovery or hemodynamic stability. These findings support its use as a viable alternative to OBA, particularly in the minimally invasive repair of the pectus excavatum.

## 1. Introduction

The minimally invasive repair of pectus excavatum (MIRPE) is widely performed in children to address progressive cardiopulmonary complications [1]. In pediatric patients, it relieves cardiopulmonary compression and enhances the long-term functional outcomes. However, in adults, pectus excavatum can still cause considerable physiological issues, including dyspnea, reduced exercise tolerance, and esthetic concerns [2]. Despite its benefits, MIRPE in adults is less common due to higher complication risks and significant postoperative pain.

Compared to pediatric patients, adults undergoing MIRPE encounter greater challenges [3]. A more rigid chest wall along with increased risks of bar displacement, bleeding, and extended recovery necessitate advanced pain management strategies. Effective postoperative pain control is essential to improving recovery quality, shortening hospital stays, and optimizing patient outcomes [4,5]. However, research on optimizing anesthetic techniques for adult MIRPE remains limited.

Opioids have long been the cornerstone of intraoperative and postoperative pain management, offering reliable analgesia and stable hemodynamics [6]. However, their use is frequently associated with a range of adverse effects, collectively termed opioid-related adverse drug events (ORADEs). Common complications include postoperative nausea and vomiting (PONV), constipation, urinary retention, pruritus, sedation, cognitive impairment, and respiratory depression, which, in severe cases, can lead to fatal outcomes [7,8]. These complications often result in prolonged hospital stays, higher healthcare costs, and decreased patient satisfaction [7,9].

Beyond these immediate risks, opioids carry significant long-term concerns, such as opioid-induced hyperalgesia, chronic pain syndromes, and dependency, even with appropriate use [10,11]. The widespread reliance on opioids in medical settings has also fueled a global health crisis, with opioid dependency and misuse now contributing significantly to morbidity and mortality worldwide [10,12]. These challenges underscore the urgent need for alternative pain management approaches that minimize opioid exposure while ensuring effective analgesia.

In response to the opioid crisis, opioid-sparing anesthesia (OSA) has garnered increasing interest [13,14]. OSA incorporates agents such as dexmedetomidine, lidocaine, ketamine, neuraxial blocks, and multimodal analgesia to minimize or eliminate opioid use while maintaining effective pain control. Among these, dexmedetomidine (DEX) has drawn particular attention for reducing opioid consumption and providing analgesia without the adverse effects typically associated with opioids [15].

DEX is a highly selective α-2 adrenergic agonist that inhibits norepinephrine release, thereby suppressing sympathetic activity in the central nervous system [16,17]. This mechanism provides dose-dependent and reversible sedation, analgesia, and sympatholytic effects. Unlike traditional sedatives, DEX induces a sedative state similar to natural sleep while preserving respiratory function, making it well-suited for light-to-moderate sedation [18,19]. Initially introduced for sedation in patients in intensive care units, DEX is widely used to manage patients who are being mechanically ventilated, offering advantages such as maintaining respiratory function, facilitating early extubation, and reducing the risk of delirium [20,21]. In anesthesiology, DEX is employed as an adjunct to primary anesthetics, neuraxial blocks, or patient-controlled analgesia (PCA) [22]. The perioperative use of DEX activates endogenous analgesic pathways and attenuates perioperative stress responses [23,24]. Through these mechanisms, it effectively lowers pain scores, reduces opioid use, and improves recovery quality [22,25,26].

Given these promising pharmacological and clinical properties, we hypothesize that implementing DEX-based OSA in MIRPE surgery will reduce opioid consumption, alleviate pain intensity, and lower opioid-related complications compared to conventional opioid-based balanced anesthesia. This study aims to compare opioid consumption between DEX-based OSA and opioid-based anesthesia (OBA) in patients undergoing MIRPE.

## 2. Materials and Methods

### 2.1. Study Population

This prospective, single-blind, randomized controlled trial was conducted following approval from the Institutional Review Board on 28 May 2019 (approval number: KC19MCSI0334) and registration on ClinicalTrials.gov on 2 September 2019 (NCT04073758). This study adhered to the ethical principles outlined in the Declaration of Helsinki, and written informed consent was obtained from all enrolled participants.

This study was conducted between September 2019 and December 2022. The inclusion criteria were as follows: age between 20 and 75 years; American Society of Anesthesiologists (ASA) physical status classification I–III; and scheduled for the elective minimally invasive repair of pectus excavatum (MIRPE). The exclusion criteria included the following: history of prior surgeries; chronic analgesic use or chronic pain; psychiatric or neurological disease; significant arrhythmia; severe cardiovascular, renal, or hepatic disorders; pregnancy or breastfeeding; known drug allergies; and refusal to participate.

### 2.2. Randomization and Blinding

Patients were randomly assigned to either the OBA group or the OFA group using a computer-generated randomization table. Due to differences in drug administration protocols, the attending anesthesiologists were aware of group assignments. However, the patients, the attending surgeons, and the medical caregiver responsible for perioperative care and outcome assessments remained blinded to group assignment throughout the study.

### 2.3. General Anesthesia and Surgical Procedure

Premedication was not administered before surgery. General anesthesia was initiated using intravenous propofol 1.5 mg/kg (Fresenius-Kabi, Bad Homburg, Germany) and rocuronium 0.8 mg/kg (Esmeron^®^, MSD Korea, Seoul, Republic of Korea), with standard monitoring which included electrocardiography, non-invasive blood pressure, pulse oximetry, oxygen saturation, end-tidal carbon dioxide (EtCO_2_), and Bispectral Index™ (BIS; Aspect Medical Systems Inc., Newton, MA, USA). After tracheal intubation, mechanical ventilation was set with a fraction of inspired oxygen (FiO_2_) of 0.5, a tidal volume of 6 mL/kg, and EtCO_2_ maintained between 35 and 40 mmHg. Continuous arterial pressure was monitored via an arterial catheter inserted into the dorsalis pedis artery. Lactated Ringer’s solution was infused at a maintenance rate of 4 mL/kg/h.

Anesthesia was sustained using 1.5–2.0 vol% sevoflurane (Sevorane^®^, AbbVie Korea, Seoul, Republic of Korea) and the assigned study medications. Adjustments to anesthetic drugs were guided by maintaining the systolic blood pressure and heart rate within 20% of the baseline values, with BIS between 40 and 60. In the OSA group, dexmedetomidine (Precedex^®^, Pfizer, New York, NY, USA) was administered as a loading dose of 0.6 μg/kg over 10 min before induction, followed by a continuous infusion at 0.5 μg/kg/h, titrated in increments of 0.1 μg/kg/h as needed. These doses were initially determined based on our institution’s thoracic surgery protocols and further refined through an internally conducted pilot study. The infusion was discontinued once wound closure began. In the OBA group, remifentanil (Ultiva^®^, GlaxoSmithKline, Brentford, UK) was delivered via a target-controlled infusion system (Orchestra Base Primea^®^, Fresenius Vial, Brezins, France) with an effect-site concentration of 3.0–4.0 ng/mL during induction and adjusted to 2.0–5.0 ng/mL during the procedure. Hypotension (systolic blood pressure <90 mmHg) was treated with ephedrine (4 mg), and bradycardia (heart rate <50 bpm) was managed with atropine (0.25 mg). Sugammadex 2–4 mg/kg (Bridion^®^, Merk & Co., Rahway, NJ, USA) was administered at the end of the procedure based on the train-of-four (TOF) ratio, followed by extubation.

All surgeries were performed by a single surgeon following the same protocol [27]. Patients were positioned supine with arms suspended overhead to optimize the surgical field. The insertion points for the pectus bars were identified based on the depressed area and hinge points bilaterally. Each bar (Pectus Bar^®^, Biomet Microfixation, Jacksonville, FL, USA) was custom-shaped during surgery to match the patient’s chest contour. Small incisions were made in the midaxillary region bilaterally, and the bars were inserted, passed under the sternum, and rotated 180° to elevate the chest wall. Stabilization was achieved using claw fixators or bridge plates, with drains placed only in cases of significant bleeding.

At the end of the procedure, intercostal nerve blocks were performed at the 4th to 9th intercostal spaces bilaterally using 2 mL of 0.5% ropivacaine per level in both groups. Additionally, a thoracic continuous wound infiltration system (CWIS; On-Q^®^ Pain Relief System, Halyard, Alpharetta, GA, USA) was positioned subcutaneously to continuously deliver 0.25% ropivacaine (Naropin^®^, Aspen Pharmacare, Durban, South Africa) at a rate of 4 mL/h. Intravenous patient-controlled analgesia (PCA) was prepared with fentanyl (20 mcg/kg) diluted in saline to a total volume of 100 mL. PCA devices (AutoMed 3200^®^, ACE Medical Corp., Ltd., Seoul, Republic of Korea) were set to deliver a basal infusion of 1 mL/h and a bolus of 1 mL, with a lockout interval of 10 min. PCA was initiated in the postoperative anesthetic care unit (PACU). Pain was assessed using a visual analogue scale (VAS) at 10 min intervals in the PACU and every 4 h in the ward. If a patient’s VAS score exceeded 4 despite PCA, rescue fentanyl (1 µg/kg) was administered, and if pain persisted, intravenous tramadol (50 mg) was given. To prevent postoperative nausea and vomiting (PONV), palonosetron (75 mcg) was administered 30 min before surgery was completed. For cases of PONV, intravenous metoclopramide (10 mg) was administered as required.

### 2.4. Outcome Measurement

The primary outcome was the cumulative analgesic consumption within the first 24 h postoperatively. This included opioids delivered through PCA and any additional analgesics administered in the ward, such as tramadol and pethidine. All administered drugs were converted into morphine-equivalent doses (MEDs) using the Opioid Analgesic Conversion Table [28], and the total was calculated in MED.

The secondary outcomes included pain intensity assessed using VAS scores at the recovery unit, and at 1–6, 6–24, and 24–48 h postoperatively. Participants noted the highest pain intensity during each period using a 100 mm VAS ruler, reflecting the most severe pain experienced since the last assessment. Additionally, total analgesic consumption, converted into MED, was measured at these same time points.

Recovery outcomes, including the time from the discontinuation of volatile anesthetics to eye opening and extubation, were used to evaluate emergence speed. The time to first rescue analgesia was also documented. Hemodynamic variables such as blood pressure and heart rate were monitored at predefined intervals: before anesthesia induction, immediately post intubation, at the time of incision, 30 min after incision, and at the conclusion of surgery. The incidence of intraoperative hypotension (systolic blood pressure <90 mmHg) and bradycardia (heart rate <50 bpm) was recorded, along with the administration frequency of rescue medications (ephedrine and atropine).

Postoperative complications related to opioids—such as PONV, hypotension, constipation, urinary retention, dizziness, respiratory depression, and temporary PCA discontinuation—were documented. Blood samples for cortisol, epinephrine, and norepinephrine levels were collected at the baseline, at the time of incision, after the pectus bar flip, and immediately after surgery. Plasma samples were obtained by centrifuging at 3000 rpm for 10 min at a temperature of 4 °C, with all specimens processed within an hour and subsequently stored at −70 °C. Epinephrine and norepinephrine levels were quantified using high-performance liquid chromatography (Agilent 1200, Agilent Technologies, CA, USA), and cortisol levels were analyzed using an electrochemiluminescence immunoassay (Cat no. 06687733, Roche Diagnostics, Mannheim, Germany).

### 2.5. Sample Size and Statistical Analysis

The sample size was determined based on prior research [16], which indicated a 30% reduction in opioid consumption with an anticipated standard deviation of 10%. To ensure 80% statistical power at a significance level of 0.05, a minimum of 38 patients per group were required. Considering an estimated dropout rate of 10%, 42 participants were recruited for each group, totaling 84 subjects for this study.

Statistical analyses were conducted using the SPSS Statistics software (version 26.0; IBM Corp., Armonk, NY, USA). Continuous variables were expressed as mean ± standard deviation (SD) and assessed using either independent *t*-tests or Mann–Whitney U tests, depending on the data distribution. Categorical variables were presented as frequencies (proportions) and analyzed using chi-square or Fisher’s exact tests, as appropriate. Changes in pain intensity and opioid consumption over time were analyzed using repeated measures analysis of variance (ANOVA). For all statistical tests, significance was defined as a two-tailed *p*-value of less than 0.05.

## 3. Results

**Figure 1:**
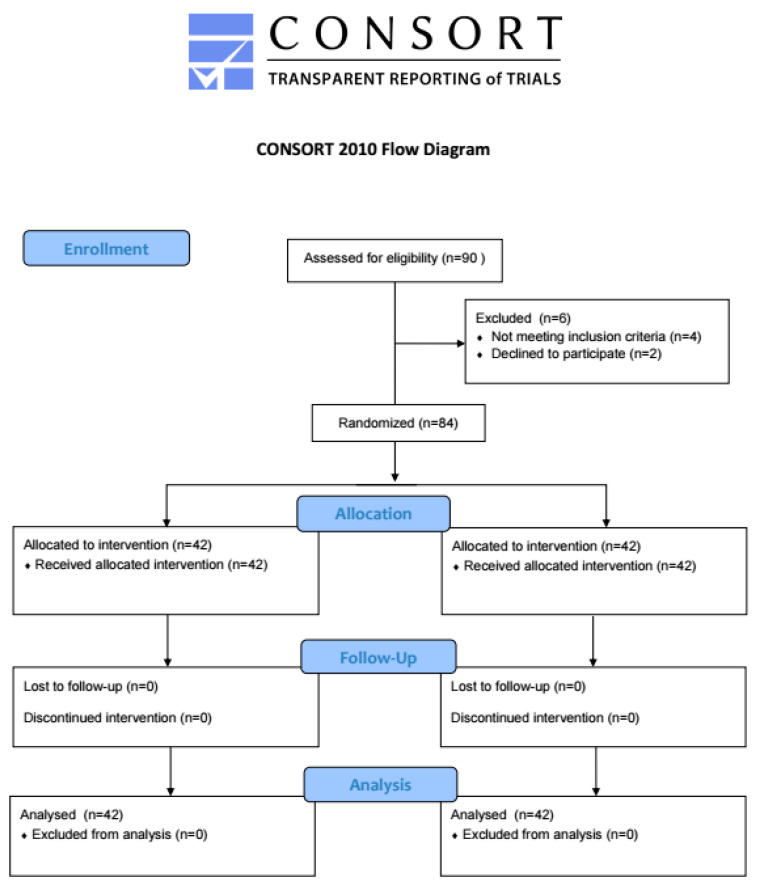
Consolidated standards of reporting trials (CONSORT) flowchart of this study.

A total of 90 participants were recruited and randomly assigned to one of the two groups, with 84 individuals completing this study (Figure 1).

No significant differences were observed between the two groups regarding the baseline characteristics, including age, sex, ASA classification, body measurements, and Haller index (Table 1). Similarly, intraoperative variables were found to be comparable across both groups (Table 1). The total amounts of remifentanil and dexmedetomidine administered in each group were 1117 ± 442 μg and 123 ± 33 μg, respectively, with mean infusion rates of 9.0 ± 4.5 μg/kg/hr for remifentanil and 0.9 ± 0.3 μg/kg/hr for dexmedetomidine. The concentration of fentanyl of IV PCA solution was comparable between the two groups, with 12.1 ± 2.0 μg/mL in the OBA group and 12.5 ± 3.2 μg/mL in the OSA group (*p* = 0.513).

Table 2 shows the postoperative pain intensity and analgesic consumption across several time intervals. The total MED administered within 24 h postoperatively, the primary outcome of this study, was considerably lower in the OSA group than in the OBA group. (55.4 ± 31.1 mg vs. 80.2 ± 26.7 mg, *p* < 0.001). The postoperative pain intensity, evaluated with the VAS, was consistently reduced in the OSA group across all measured time points. The OSA group also demonstrated significantly lower fentanyl consumption via intravenous PCA, and the total MED over 48 h was similarly reduced in the OSA group. Additionally, fewer rescue analgesics were required in the OSA group, with a significant difference in the recovery unit (*p* = 0.002).

**Figure 2:**
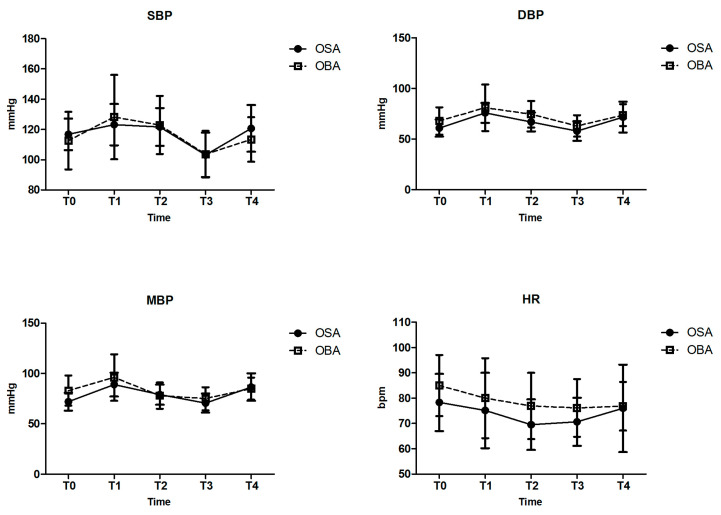
Intraoperative hemodynamic parameters. T0: before anesthesia induction; T1: immediately after tracheal intubation; T2: at the time of incision; T3: 30min after incision; T4: at the end of surgery; SBP: systolic blood pressure; DBP: diastolic blood pressure; MBP: mean blood pressure; and HR: heart rate.

Both groups maintained stable intraoperative hemodynamics, with no significant differences in blood pressure or heart rate at predefined time points (Figure 2). The incidence of hypotension was 54.8% in the OBA group and 42.9% in the OSA group (*p* = 0.383), while bradycardia occurred in 54.8% of the OBA group and 52.4% of the OSA group (*p* = 1.000), showing comparable rates between the two groups. Rescue interventions, including ephedrine and atropine administration, were required with similar frequency in both groups (Table 3).

The postoperative recovery outcomes, including the time to eye opening, extubation, and first rescue analgesia, showed no significant differences between the groups (Table 3). The total recovery duration was also comparable, with no statistically significant differences. Adverse events were rare in both groups. Six patients in the OBA group and five patients in the OSA group experienced PONV, without significant differences between the groups (*p* = 1.000). Constipation occurred in one patient from each group, and PCA was withdrawn in one patient from the OBA group due to PONV. No other opioid-related complications were reported.

**Figure 3:**
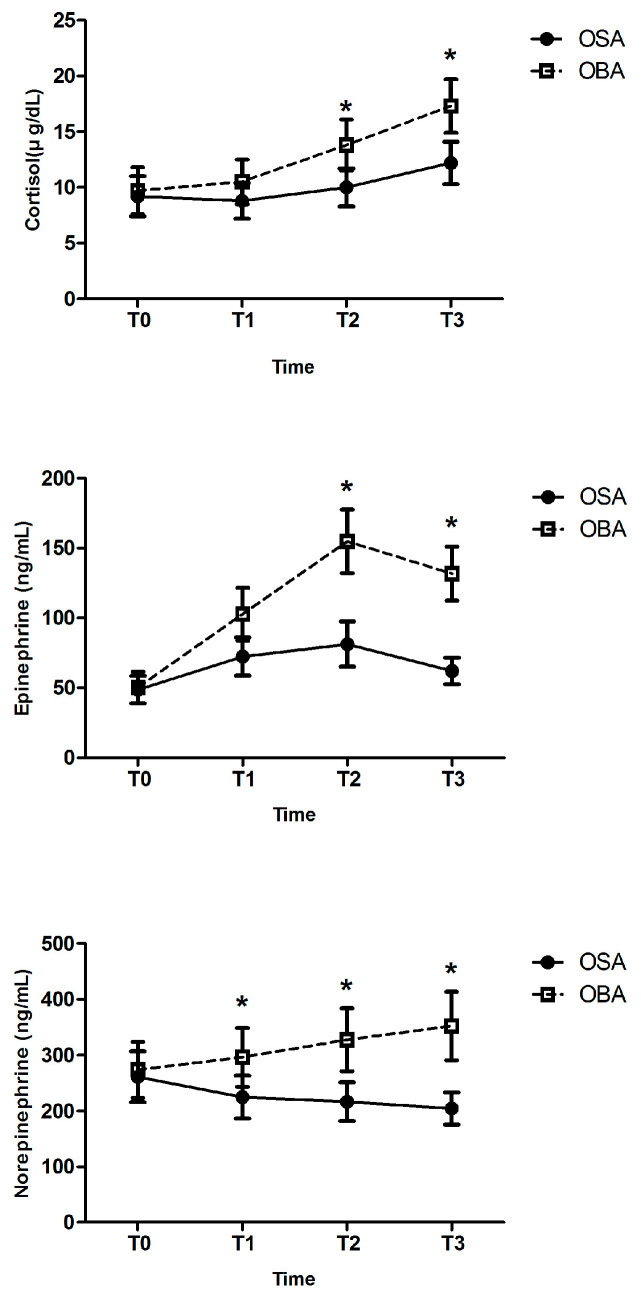
Perioperative stress hormone levels. T0: baseline; T1: at the time of incision; T2: after pectus bar flip; and T3: immediately after surgery. * *p* < 0.05 between two groups.

Perioperative stress hormone levels, measured at the baseline, the time of incision, after the pectus bar flip, and immediately after surgery, showed that both groups experienced an increase in cortisol, epinephrine, and norepinephrine during surgery (Figure 3). However, the OSA group showed a smaller increase in epinephrine, with an insignificant decrease in norepinephrine, suggesting attenuated sympathetic activation. Notably, epinephrine levels peaked in both groups at the time of the pectus bar flip, although the OFA group showed a significantly lower peak compared to the OBA group. Cortisol secretion also increased continuously in both groups, but the OSA group demonstrated a more attenuated increase following DEX infusion, resulting in a significant difference between the two groups.

## 4. Discussion

This study aimed to investigate the influence of DEX-based OSA on postoperative opioid consumption in patients undergoing MIRPE. The findings demonstrated a significant reduction in both pain intensity and analgesic consumption within the first 48 h postoperatively in the OSA group. No significant differences were observed between the OSA and OBA groups concerning opioid-related complications, intraoperative hemodynamics, or recovery times.

Our findings are consistent with those of previous studies demonstrating the benefits of DEX-based OSA across various types of surgeries. In gynecological laparoscopy, DEX-based OSA was associated with reduced pain intensity, lower analgesic consumption, and fewer opioid-related side effects, with no significant differences in recovery times compared to traditional OBA [29]. Similarly, OSA protocols have been shown to improve quality of recovery (QoR) scores (QoR-40) and reduce pain scores, alongside decreased levels of stress hormones [30]. In laparoscopic cholecystectomy, DEX exhibited immunomodulatory effects by reducing IL-6 levels while simultaneously decreasing opioid consumption [31,32]. In bariatric surgery, the use of DEX was linked to reductions in PONV, pain scores, and analgesic requirements [33,34]. In major spine surgeries, intraoperative DEX effectively attenuated stress responses, maintained hemodynamic stability, and lowered the postoperative pain scores [35,36]. In video-assisted thoracic surgery (VATS), OSA protocols incorporating DEX significantly reduced morphine consumption and improved both QoR-40 scores and pain outcomes, while maintaining stable hemodynamics [37,38]. Collectively, these studies suggest that OSA protocols using DEX offer consistent benefits across various surgical settings, including reduced opioid use, enhanced recovery quality, and improved pain control, without compromising safety or hemodynamic stability. Beyond DEX-based protocols, recent studies have demonstrated the broader benefits of OSA across various surgical contexts. Several meta-analyses reported that OSA significantly reduced PONV and reduced postoperative opioid consumption while maintaining effective analgesia [39,40]. Similarly, Piccioni et al. found that OSA in thoracic surgeries resulted in lower opioid consumption, improved pain scores, and fewer complications at 48 h postoperatively [41]. These findings align with our study, emphasizing that OSA can optimize perioperative pain management, reduce opioid-related adverse effects, and improve overall recovery quality.

In contrast to our findings, several studies have reported no superior recovery outcomes in DEX-based OSA. In gynecological laparoscopy, Massoth et al. [42] found no significant reduction in PONV, pain scores, or opioid consumption when comparing DEX-based OSA with sufentanil-based OBA. Similarly, another study applying OSA within an ERAS protocol for gynecological surgery reported non-inferior pain outcomes but noted delayed emergence and reduced PONV with OSA [43]. In thoracic surgeries, propensity-matched studies involving intraoperative DEX infusions found no differences in pain scores or opioid use throughout the hospital stay [44,45]. These inconsistent findings suggest that the variability in DEX-based OSA outcomes may be attributed to differences in administration protocols, such as the use of adjunct medications, DEX dosage, timing of infusion, and patient populations. Future studies should aim to standardize OSA protocols and explore optimal dosing regimens to maximize the benefits of DEX while minimizing potential side effects.

While our study did not directly investigate the underlying mechanisms, we propose two potential explanations for the observed reduction in opioid consumption between the OBA and OSA groups. First, opioid-induced hyperalgesia (OIH) may have contributed to the higher opioid requirements in the OBA group due to the use of remifentanil. Previous studies have shown that remifentanil, a potent and short-acting opioid widely used in surgery, induces acute opioid tolerance and hyperalgesia by activating NMDA receptors and oxidative stress pathways [46,47]. These mechanisms can lead to heightened pain sensitivity, necessitating greater postoperative opioid consumption. Notably, even brief infusions of remifentanil (e.g., 90 min) can trigger acute tolerance, with higher doses making patients more susceptible to OIH. By avoiding remifentanil in the OSA group, this effect may have been mitigated, contributing to the observed reduction in postoperative opioid use. Second, the intrinsic analgesic properties of DEX likely played a role. As a selective alpha-2 adrenergic receptor agonist, DEX reduces sympathetic outflow and suppresses norepinephrine release, leading to analgesic and stress-relieving effects [17]. This sympatholytic mechanism not only enhances multimodal analgesia but also aligns with the lower levels of stress hormones observed in the OSA group. By mitigating the physiological stress response, DEX likely contributed to reduced pain perception and opioid demand during the postoperative period. These findings highlight the clinical value of DEX-based OSA as a viable alternative to remifentanil, particularly in surgeries requiring prolonged opioid use, by reducing hyperalgesia, stress responses, and opioid-related side effects.

Contrary to expectations, our study found no difference in opioid-related complications, such as PONV, between the OSA and OBA groups. This contrasts with previous meta-analyses [48,49], which reported that intraoperative opioid avoidance significantly reduces PONV. A possible explanation for this discrepancy is the relatively low opioid consumption in both groups, driven by the multimodal analgesia protocol employed, including a CWIS, which reduced opioid use by nearly 60% in MIRPE surgeries at our institution. Additionally, the routine administration of prophylactic antiemetics before the end of surgery likely contributed to the low incidence of PONV. The lower proportion of female participants, who are more susceptible to PONV, may also have influenced the lack of difference in outcomes.

The prolonged analgesic effects of DEX observed in our study, extending up to 48 h postoperatively, raise important questions about its underlying mechanisms. While the elimination half-life of DEX is relatively short (2–2.5 h) [16,17], its analgesic effects appear to extend beyond its immediate pharmacokinetics. This discrepancy may be explained by two potential mechanisms. First, DEX exerts its analgesic effects primarily through alpha-2 adrenergic receptor activation, which modulates central and peripheral pain pathways [16,17]. Unlike its sedative effects, the analgesic action of DEX may involve distinct downstream signalling pathways with a prolonged duration. Second, DEX is known to enhance the efficacy of co-administered analgesics, including local anesthetics and opioids [50]. In our study, this effect likely potentiated the analgesic action of intercostal block- and CWIS-administered local anesthetics, as well as fentanyl delivered via intravenous PCA in the immediate postoperative period. Supporting evidence from previous studies further corroborates this hypothesis. In patients undergoing pulmonary resection, intraoperative DEX loading improved both the pain domain and overall QoR-40 scores up to postoperative day (POD) 2 [51]. Similarly, in colectomy patients, continuous intraoperative DEX infusion resulted in reduced VAS pain scores and opioid consumption at 24 h postoperatively, along with sustained improvements in QoR-40 scores up to PODs 3 and 7 [52]. These findings suggest that DEX may have a synergistic effect with other analgesic agents, contributing to prolonged postoperative pain relief. However, further studies are necessary to elucidate the exact mechanisms underlying these prolonged effects and explore the potential for optimizing multimodal analgesia protocols incorporating DEX.

Despite concerns that DEX may prolong recovery and cause hemodynamic instability [17,23], our study found no significant difference between the OSA and OBA groups in postoperative recovery time or intraoperative hemodynamic stability. DEX reduces sympathetic outflow from the locus ceruleus, decreasing norepinephrine release and activating parasympathetic pathways. While these effects can cause sedation and bradycardia, they appear to be dose-dependent, with higher doses linked to prolonged recovery and more profound hemodynamic changes. In our study, the use of a moderate loading dose followed by a controlled maintenance infusion (below 1 μg/kg/h) likely mitigated these effects, aligning with prior findings that lower DEX doses maintain stable hemodynamics without compromising recovery [33,37]. The absence of significant bradycardia or hypotension in our study reinforces the safety of this dosing regimen in procedures like MIRPE, particularly in relatively healthy patients without cardiovascular comorbidities. Previous studies have reported severe bradycardia with higher DEX doses, leading to early trial termination in some cases [53]. The stable hemodynamics and comparable recovery times observed in our study suggest that this dosing protocol may represent an optimal approach for DEX-based OSA in minimally invasive thoracic surgeries.

This study has several limitations. First, the attending anesthesiologists were not blinded to the group allocation due to protocol differences between the two groups. This partial blinding may have introduced bias in intraoperative management, particularly in drug administration and monitoring. However, all other clinical staff involved in patient care, including surgeons and postoperative care teams, were blinded throughout the study to minimize potential bias. Second, the evaluation period was limited to 48 h postoperatively, during which the same multimodal analgesia protocol was applied to both groups. This short follow-up restricted our ability to assess longer-term outcomes. While no significant differences in short-term recovery were observed, previous studies on VATS suggest that DEX-based OSA may influence chronic pain syndromes rather than early recovery outcomes [45]. Therefore, future studies with extended follow-up durations are needed to explore the potential long-term benefits of OSA on postoperative pain and recovery. Third, the attending surgeon prioritized intercostal block combined with CWIS as the primary pain management strategy. Consequently, preoperative regional anesthesia techniques, such as erector spinae plane block or paravertebral block, were not utilized due to concerns about the cumulative dose of local anesthetics. Additionally, given the severity of postoperative pain, PCA with fentanyl was employed despite the use of non-opioid medications and CWIS. These factors limited our ability to fully evaluate a true opioid-free anesthesia protocol. Future studies should explore the combined use of preoperative regional anesthesia techniques and DEX to better assess the feasibility and efficacy of opioid-free anesthesia in surgeries associated with severe pain. Lastly, a limitation concerns the precise evaluation of DEX’s mechanism in reducing OIH. DEX is believed to mitigate OIH by modulating reactive oxygen species production and NMDA receptor activity in the central nervous system. Although preclinical studies have shown that DEX suppresses oxidative stress pathways and NMDA receptor activation, these findings are based on spinal cord samples from animal models [54,55]. In our study, ethical and practical constraints precluded the collection of spinal samples, limiting our ability to directly confirm these central mechanisms.

## 5. Conclusions

In conclusion, our study demonstrated that DEX-based OSA significantly reduced postoperative pain intensity and opioid consumption within 48 h after MIRPE. Despite concerns about hemodynamic instability and delayed recovery with DEX, our results showed comparable recovery times and stable intraoperative hemodynamics between the OSA and OBA groups. These findings support the use of DEX-based OSA as a safe and effective alternative to OBA, ensuring effective pain management while reducing opioid consumption and the associated adverse effects. Further studies are necessary to corroborate these findings and investigate the potential long-term benefits of OSA across different surgical environments.

## Figures and Tables

**Table 1:** Patient demographics and intraoperative variables.

	OBA Group (N = 42)	OSA Group (N = 42)	*p*
Age (yr)	23.8 ± 4.6	23.7 ± 2.7	0.954
Sex (male/female)	35/7	33/9	0.781
ASA class (1/2)	38/4	36/6	0.736
Height (cm)	172.5 ± 9.6	173.1 ± 7.2	0.763
Weight (kg)	61.2 ± 11.6	59.7 ± 12.3	0.585
Haller index	5.2 ± 1.1	5.0 ± 1.2	0.584
Number of pectus bars (2/3)	34/8	33/9	1.000
Operation time (min)	135.1 ± 40.9	136.4 ± 47.8	0.897
Anesthesia time (min)	174.3 ± 32.2	180.9 ± 39.1	0.407
Sevo (vol%)	1.8 ± 0.3	1.7 ± 0.3	0.257
Remifentanil infusion rate (μg/kg/h)	9.0 ± 4.5	N/A	
Dexmedetomidine infusion rate (μg/kg/h)	N/A	0.9 ± 0.3	

Values are expressed as a number or mean ± standard deviation, as appropriate. Sevo: intraoperative mean concentration of sevoflurane.

**Table 2:** Postoperative pain intensity and analgesic consumption.

	OBA Group (N = 42)	OSA Group (N = 42)	*p*
At recovery unit			
Highest VAS	8.0 ± 2.1	4.9 ± 1.5	<0.001
PCA consumption (mL)	6.2 ± 2.6	3.4 ± 1.5	<0.001
Rescue requirement (0/1/2)	2 / 36 / 4	14 / 25 / 3	0.002
Rescue opioid (μg)	65.0 ± 30.0	41.7 ± 34.8	0.001
MED (mg)	14.0 ± 4.4	10.8 ± 5.2	0.003
1–6 h after surgery			
Highest VAS	7.1 ± 1.9	5.3 ± 1.8	<0.001
PCA consumption (mL)	17.8 ± 7.7	12.9 ± 7.2	0.003
Rescue requirement (0/1/2)	16/19/7	24/15/3	0.178
Rescue opioid (μg)	16.9 ± 15.7	11.4 ± 13.4	0.089
MED (mg)	23.2 ± 10.1	17.6 ± 11.2	0.019
6–24 h after surgery			
Highest VAS	5.4 ± 2.1	3.9 ± 1.5	<0.001
PCA consumption (mL)	33.8 ± 18.8	20.3 ± 14.6	<0.001
Rescue requirement (0/1/2/3)	17/17/6/2	21/18/3/0	0.327
Rescue opioid (μg)	16.8 ± 15.0	12.9 ± 13.0	0.204
MED (mg)	43.0 ± 25.5	27.0 ± 22.8	0.003
24–48 h after surgery			
Highest VAS	4.6 ± 2.2	3.6 ± 1.7	0.018
PCA consumption (mL)	43.5 ± 15.5	33.1 ± 19.0	0.007
Rescue requirement (0/1/2)	17 / 21 / 4	27 / 9 / 6	0.027
Rescue opioid (μg)	21.2 ± 27.0	9.6 ± 13.2	0.001
MED (mg)	55.6 ± 22.1	41.1 ± 23.6	0.005

The values are expressed as numbers (proportions) or mean ± standard deviation, as appropriate. VAS: visual analogue scale; PCA: patient-controlled analgesia; and MED: morphine-equivalent dose.

**Table 3:** Recovery characteristics and perioperative adverse events.

	OBA Group (N = 42)	OSA Group (N = 42)	*p*
At recovery unit			
Time to eye opening (min)	3.5 ± 1.3	4.1 ± 1.8	0.085
Time to extubation (min)	5.6 ± 2.0	6.5 ± 2.8	0.066
Time to first rescue (min)	12.3 ± 7.6	13.4 ± 14.3	0.663
Total recovery duration (min)	54.0 ± 14.6	57.7 ± 15.4	0.257
Intraoperative adverse events			
Hypotension (Y)	23 (54.8%)	18 (42.9%)	0.383
Rescue ephedrine (Y)	21 (50.0%)	15 (35.7%)	0.270
Ephedrine dose (mg)	0.5 ± 0.5	0.6 ± 0.5	0.190
Bradycardia (Y)	23 (54.8%)	22 (52.4%)	1.000
Rescue atropine (Y)	18 (42.9%)	19 (45.2%)	1.000
Atropine dose (mg)	0.6 ± 0.5	0.6 ± 0.5	0.829
Postoperative adverse events			
PONV	6 (14.3%)	5 (11.9%)	1.000
Constipation	1 (2.4%)	1 (2.4%)	1.000

Values are expressed as a number (proportion) or mean ± standard deviation. PONV: postoperative nausea and vomiting.

## Data Availability

The data generated in this study can be shared upon reasonable request to the corresponding author.
